# Effects of the Processing Parameters on the Shear Viscosity of Cyclic Olefin Copolymer Plasticized by Ultrasonic Vibration Energy

**DOI:** 10.3390/polym12030539

**Published:** 2020-03-02

**Authors:** Jin Lu, Yuanbao Qiang, Wangqing Wu, Bingyan Jiang

**Affiliations:** 1 State Key Laboratory of High Performance Complex Manufacturing, Central South University, Lushan South Road 932, Changsha 410083, China; jinloo@csu.edu.cn (J.L.); yuanbaoqiang@csu.edu.cn (Y.Q.); jby@csu.edu.cn (B.J.); 2 School of Mechanical and Electrical Engineering, Central South University, Lushan South Road 932, Changsha 410083, China

**Keywords:** ultrasonic plasticization, COC polymer, shear viscosity, microinjection molding, rheology

## Abstract

Shear viscosity of the cyclic olefin copolymer (COC) plasticized by ultrasonic vibration energy is characterized by high pressure capillary rheometer. Two different plasticization modes were adopted to prepare the samples with an in-house developed prototype machine. Single-factor experiments were conducted to investigate the effects of ultrasonic energy on the shear viscosity. The influences of the processing parameters and the plasticization modes were analyzed and compared. The results showed that the shear viscosity of COC was reduced under various parameter combinations, and demonstrated a significant difference in the lower shear rate range in comparison with the control samples; the results of gel permeation chromatography (GPC) showed that the COC’s number average molecular weight (${\bar{M}}_{n}$) was decreased and the polymer dispersity index (*PDI*) was increased due to the plasticization by ultrasonic vibration energy. This could be further account for the decrease of the shear viscosity of COC. Moreover, the predominant ultrasonic parameter changed in different modes of plasticization according to the statistical analysis based on the Statistical Product and Service Solutions (SPSS) software.

## 1. Introduction

So far, microinjection molding has been successfully and widely applied in the fabrication of polymeric micro parts [1], such as micro heat exchangers, bio-medical chips and miniaturized optical devices [2], due to its intrinsic advantages in cost saving and efficiency increasing. In addition, its ability to molding parts with considerable accuracy and excellent replicability makes it even more attractive [3]. However, as the demand for micro-parts are increasingly expanded, microinjection molding encounters new technological limitations. One of them is that higher levels of process parameters such as injection pressure and melt temperature are usually essential for an improved molding quality. This is quite energy-consuming because a large part of the energy is used to maintain the melt temperature and to drive the injection unit. Another disadvantage lies in the extremely low utilization ratio of the polymer melt. It is common that the amount of polymer plasticized by conventional screw greatly exceeds the amount needed for molding micro-parts in each cycle, due to the technical difficulty of decreasing the outer diameter of the screw, especially in some advanced applications [4]. Furthermore, due to the size effects existing in the filling process of micro-cavities, the filling process of microinjection molding is rendered extremely complicated.

In view of the challenges mentioned above, in 2002, W. Michaeli et al. [5] originally came up with an entirely new ultrasonic plasticization concept for microinjection molding. It was found that the energy consumption was much lower and the sprue of the PMMA micro-parts could be greatly reduced. In 2006, W. Michaeli et al. [6] further confirmed that a significant reduction of the cycle time can be achieved by integrating the ultrasonic plasticization into the microinjection molding process. Since then, the new ultrasonic plasticization concept has aroused widespread attention and has been believed to be an excellent potential solution for the current technological limitations of microinjection molding. To distinguish it from conventional microinjection molding (CMIM) based on screw plasticization unit, the nomenclature of ultrasonic plasticization microinjection molding (UPMIM) was proposed.

To gain a more profound understanding of the UPMIM process, Matías Sacristán et al. [7] conducted relevant research on the effects of various process parameters on the materials physical and chemical properties, microscopical morphology and mechanical performance of polylactide (PLA) tensile parts. It was found that the ultrasonic parameters had significant impacts on the morphology of the PLA tensile parts. The molecular degradation could be alleviated, even avoided by appropriate parameter combinations. Grabalosa et al. [8] and Sanchez et al. [9] concluded that the filling rate could be ameliorated by increasing ultrasonic action time, plasticizing time and plasticizing pressure. Bingyan Jiang et al. [10] studied the effects of ultrasound and temperature on the fluidities of different polymers (PMMA, PP, PA66) under micro-scale. Their research showed that by increasing ultrasound amplitude, ultrasonic action time, plasticizing pressure and mold temperature, the fluidity of both amorphous and semi-crystalline polymers could be notably improved.

As a critical parameter of polymer melt controlled by temperature, molecular properties and shear rate, the shear viscosity governs the filling process and ultimate molding quality in either CMIM or UPMIM. There have been numerous relevant studies carried out in the field of CMIM [11,12,13,14,15,16] or CMIM assisted by some other physical fields (magnetism [17] and mechanical vibration filed [18]). Although the eventual goal of UPMIM is similar to CMIM, some key differences should not be neglected. The polymer melts in CMIM are commonly generated by the compaction and shear of the rotating screw in the heated barrel, and the melt temperature can be precisely defined by the temperature control system. However, the external heating of the barrel is not necessary in UPMIM because the polymers can be plasticized merely by the ultrasonic energy in a very short time. The temperature of polymer melt is not a definable constant anymore, but possibly a function of UPMIM process parameters. In addition, the molecular chain structure of the polymer could be changed due to the intensive high energy input via high frequency ultrasonic vibration. Therefore, it is essential to investigate the mechanisms of influencing the process parameters on the shear viscosity of the polymer melt, especially for the development of a new UPMIM process. 

Up to now, there have been many studies toward the effects of ultrasound on the flowability and rheological behaviors of polymers [19,20,21,22,23,24,25]. For example, Kim et al. [19] applied ultrasound in the extrusion process of polypropylene (PP). It was found that the shear viscosity of the PP melt decreased due to the fact that the molecular chains of PP were broken by the ultrasonic vibration. Heng Lin et al. [20] studied the shear viscosity of PP and polyamide (PA66) polymers in extrusion and found that the shear viscosity of PP decreases with increasing ultrasound amplitude. The shear viscosity of PA66 increases up to a peak value and then decreases with increasing ultrasound amplitude. In these studies, the ultrasound merely plays an auxiliary role; the polymeric melts studied were generated by screw extruder and went through completely different plasticization and thermal history in comparison with the one in UPMIM. As a result, the mechanisms influencing the process parameters on the shear viscosity of polymer melt might not be the same as well.

The objective of this paper is to characterize the shear viscosity of polymer melt in the UPMIM process considering the influences of the process conditions. Cyclic olefin copolymer (COC) was selected to be studied because of its promising application in micro optical devices and microfluidic chips. With in-house developed prototype machine, the UPMIM process window for COC was determined under two different conditions; i.e., Mode 1—“sole plasticization” and Mode 2—“plasticization plus extrusion,” depending on the process with or without polymer melt extrusion. A series of parameter combinations was designed to prepare samples used in rheological measurements which were conducted on a commercial high-pressure capillary rheometer. Additionally, GPC tests were carried out to reveal the underlying mechanism which could be responsible for the change of the shear viscosity of COC. At last, commercial software for data analysis—Statistical Product and Service Solutions (SPSS) was used to quantitatively ascertain how much each parameter affects the shear viscosity of the polymer melt and to visualize the distinctions between the two modes.

## 2. Experimentation

### 2.1. Materials

A pellet-shape COC (TOPAS 5013-10L, applied in optics, produced by TOPAS^®^, Fuji, Shizuoka-ken, Japan), which is characterized by the properties shown in Table 1, is used in this study. COC was dried at 80 °C for 6 h in dryer before further utilization in case that moisture absorbed in raw material would cause negative effects on experiment.

### 2.2. Equipment for Ultrasonic Plasticization

**Figure 1:**
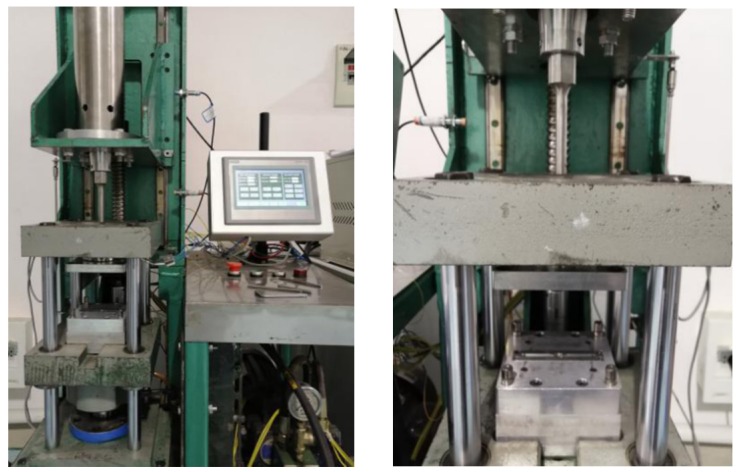
Prototype machine for ultrasonic plasticization.

**Figure 2:**
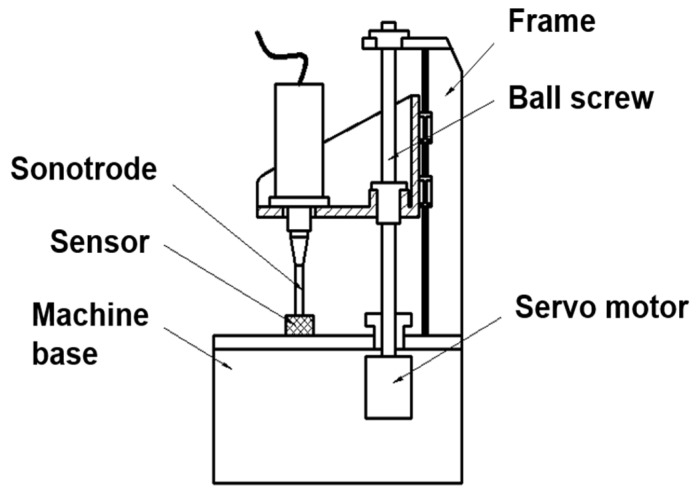
Method for determining plasticizing pressure.

An in-house developed prototype machine, as shown in Figure 1, was employed to determine the UPMIM process window for COC and to prepare the samples needed for the rheological measurements. The prototype machine, whose main characteristics are as shown in Table 2, comprises an ultrasonic vibration system, a servo motion-controlled injection unit and a hydraulic clamping system. The UPMIM process parameters can be adjusted and controlled by an in-house developed control system based on industrial programmable logic controller (PLC). The ultrasonic amplitude can be regulated via the percentage of power input. The corresponding relationship between the ultrasonic amplitude and the power input is as shown in Table 3. The plasticizing pressure was determined via the experimental setup shown in Figure 2. A load sensor was used to calibrate the specific plasticizing pressure corresponding to the torque of the servo motor.

### 2.3. Principles of Two Different Modes of Plasticization

**Figure 3:**
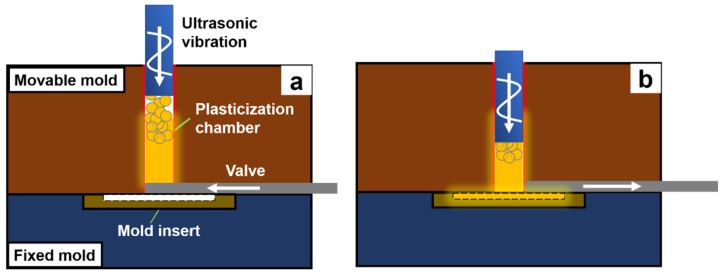
Schematic illustration of two different modes. (**a**) Mode 1, (**b**) Mode 2.

Two different modes of plasticization are used in our experiment, as shown in Figure 3. The first one is defined as “sole plasticization.” For Mode 1, only plasticization exists. And melted polymer is retained in the plasticization chamber during the whole plasticization. The second one is defined as “plasticization plus extrusion.” For Mode 2, the generated polymeric melt would be extruded out promptly from the material system during the plasticization procedure. 

### 2.4. Development of the Process Window

**Figure 4:**
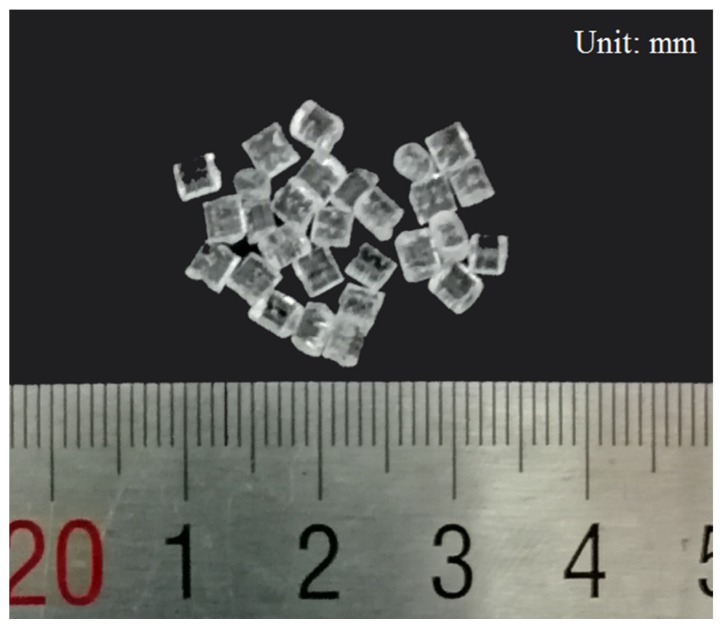
The amount of raw COC plasticized each time.

Considering no references for determining plasticizing parameters of COC, the scope of process parameters in which COC can be plasticized should be figured out; the primary ultrasonic parameters involved in our experiments are ultrasound amplitude, ultrasonic action time and plasticizing pressure. In this experiment, 100 different parameter combinations were investigated under each mode. To avoid experimental occasionality, the plasticization experiment for each parameter combination was repeated three times and raw COC of more or less than 0.35 g was plasticized each time, as shown in Figure 4.

### 2.5. Measurements

Shear viscosities of raw COC and the ultrasonically-processed variants were measured by a high pressure capillary rheometer—Göttfert RG50 with a measurement shear rate range from 10^−1^ s^−1^ to 10^5^ s^−1^ and a maximum operation temperature of 400 °C.

After acquiring the parameter scope suitable for plasticizing raw COC, some combinations were selected for preparing samples used in viscosity measurement. As to the selection of parameters, the respective principles of both the single factor experiment and energy saving were taken into account. The ultimate parameter combinations are listed in Table 4. Shear viscosity measurements were performed by a high pressure capillary rheometer; the testing temperature was 240 °C; the testing shear rate ranged from 70 s^−^^1^ to 5000 s^−^^1^; and a die with a length-to-radius ratio of 5/0.5 was used with the consideration of the size characteristics of CMIM.

To explore ultrasonic effects on molecular properties of COC, GPC measurements were conducted. Molecular weight was estimated by gel permeation chromatography (GPC) using liquid chromatography equipment (Angient, model PL-GPC 220). The polymer was dissolved and eluted in trichlorobenzenes (TCB) solvent stabilized with 2,6-di-tert-butyl-p-cresol (BHT, 0.0125%) at a flow rate of 1.00 mL/min (injected volume 200 μL, sample concentration 1.0 mg/mL). The average molecular weights and molar-mass dispersity were calculated from five different samples and polystyrene standards were used in the GPC test.

### 2.6. Statistical Analysis

Only plotting apparent viscosity-shear rate curve could not visually present the influence of ultrasound energy on shear viscosity of raw material. Hence, aside from describing shear viscosity of processed COC graphically, some statistical parameters were introduced for quantifying the effects exerted on shear viscosity by ultrasonic energy. Herein, two indices were used for quantifying ultrasonic effects; namely, decreased percentage of viscosity value—${DPV}_{\dot{\gamma },T}$ and standardized regression coefficient—*β*. ${DPV}_{\dot{\gamma },T}$ is used to measure the disparity in viscosity value between processed COC and raw COC. It is calculated by following equation:

(1) $${DPV}_{\dot{\gamma },T}=\frac{\eta_{r|\dot{\gamma },T}-\eta_{p|\dot{\gamma },T}}{\eta_{r|\dot{\gamma },T}}\times 100\%$$

where ${DPV}_{\dot{\gamma },T}$ refers to decrease percentage of viscosity value of designated shear rate and temperature; $\eta_{r|\dot{\gamma },T}$ means the viscosity value of a raw material obtained under a certain shear rate and measurement temperature, and $\eta_{p|\dot{\gamma },T}$ means the same for the processed material. Standardized regression coefficient *β* is applied to evaluate how significantly each ultrasonic parameter affects viscosity of raw material. *β* is calculated via multiple linear regression analysis (MLRA) which could be completed by SPSS. According to basic principles of MLRA, the higher the absolute value of standardized regression coefficient *β* is, the more significant the influence of corresponding independent variable on dependent variable will be.

In this work, the main independent variables are ultrasound amplitude, ultrasonic action time and plasticizing pressure, and the dependent variable is the shear viscosity of each processed material; however, viscosity value could not directly reflect the intensity of ultrasonic effects on original viscosity of raw material. Thus, ${DPV}_{\dot{\gamma },T}$ may be an appropriate choice for dependent variable.

## 3. Results

### 3.1. Preliminary Results of Processing Window

**Figure 5:**
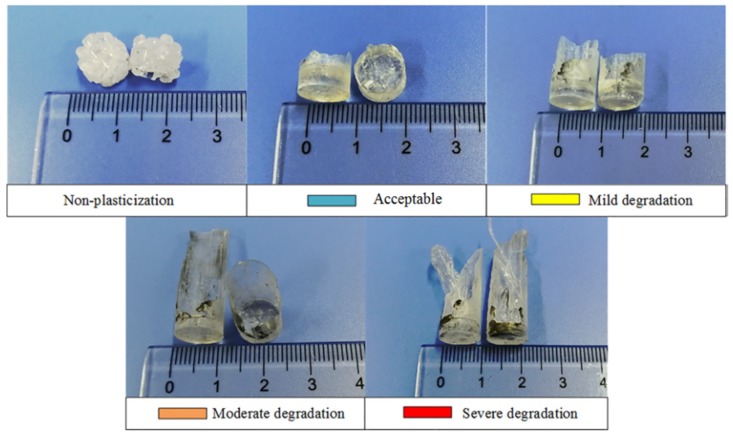
Representative samples obtained under Mode 1.

**Figure 6:**
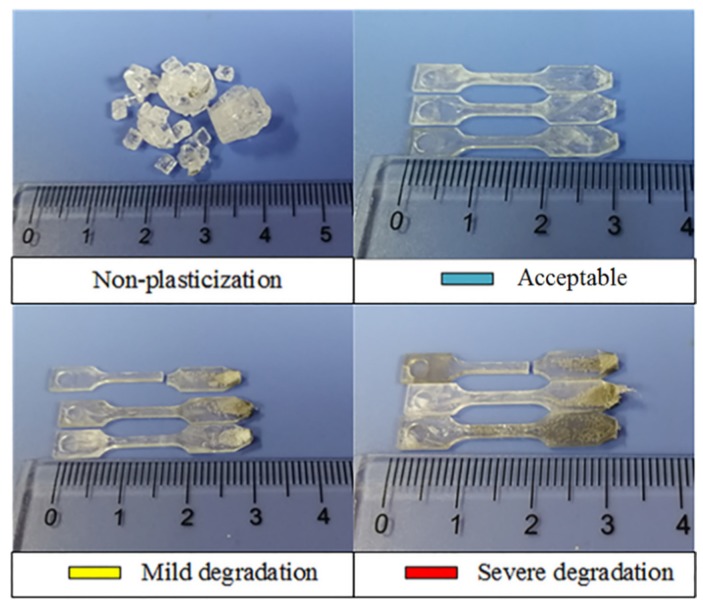
Representative samples obtained under Mode 2.

According to preliminary results, the combination under which raw COC was successfully plasticized is marked with “*”; the non-plasticized was marked with “**-**“; furthermore, those combinations succeeding in plasticizing COC but leading to degradation are painted with different colors in Table 5: yellow means mild degradation (only a quite small amount of transparent material processed blacks), orange symbolizes the moderate (appreciable blacking), red stands for the severe (appreciable blacking and sticking onto the sonotrode occur at the same time) and no degradation was painted blue. And some representative samples plasticized by ultrasonic energy under two different modes are shown in Figure 5 and Figure 6 respectively.

As shown in Table 5, all the parameter combinations for which ultrasound action time is set as 2 s fail to plasticize COC, mainly because the action time of ultrasound wave is too short to generate adequate heat for melting COC, and the heat is attributed to the combined contribution of frictional heating effects and viscoelastic heating effects [26,27]. COC could be melted by the remaining combinations; and as one certain parameter increases, such as amplitude, time or pressure, material degradation is inevitable. At least from the results of preliminary experiment, the gross parameter scope for plasticizing COC pellets was obtained.

### 3.2. Shear Viscosity Properties under Two Different Modes

In this section, shear viscosities of COC modified under two different modes are illustrated and analyzed in comparison with raw COC.

#### 3.2.1. Effects of Ultrasonic Amplitude

**Figure 7:**
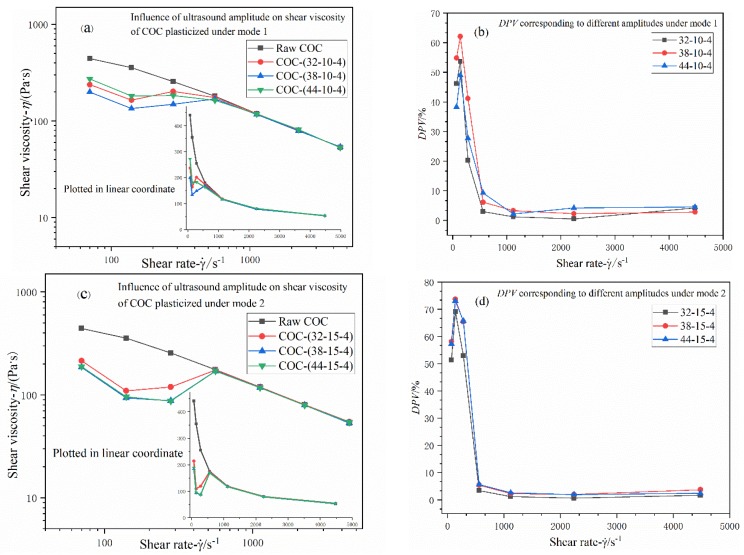
(**a**,**c**) Influence of adjusting ultrasound amplitude on shear viscosity of COC plasticized under Mode 1 and Mode 2; (**b**,**d**) *DPVs* corresponding to different ultrasound amplitudes under two different modes. Nomenclature: ultrasonic amplitude (μm); plasticizing pressure (MPa); ultrasonic action time (s).

As shown in Figure 7, under Mode 1, it is evident that ultrasound can influence the shear viscosity of COC, but there is an obvious disparity between the apparent viscosity-shear rate curve of raw material and the processed. As can be seen in Figure 7a, in low shear rate region (70–560 s^−^^1^), the viscosity values of COC processed by ultrasound with different amplitudes are considerably lower than those of raw material, and the maximum *DPVs* corresponding to three different amplitudes are 53% (32 μm), 62% (38 μm) and 49% (44 μm) respectively, at the shear rate of 140 s^-1^ according to Figure 7b. In high shear rate range (1120–5000 s^−1^), the viscosity values of processed material are almost equal to those of the non-processed. It is noteworthy that *DPVs* corresponding to these three amplitudes all tend to remain nearly unchangeable as shear rates increase. 

Similarly, viscosity curves of COC processed by different amplitudes under Mode 2 are quite similar to those of Mode 1, but there are still some noticeable discrepancies. In the low shear rate region (70–560 s^−1^), viscosity values of processed materials are all lower than the raw presented, and decrease with shear rate in an undulant trend simultaneously. The maximum *DPVs* corresponding to three different amplitudes are 69% (32 μm), 74% (38 μm) and 73% (44 μm) respectively at the shear rate of 140 s^−1^ according to Figure 7d. However, as shown in Figure 7c, the differences lie in that viscosity values of different amplitudes are considerably closer to each other, and increasing amplitude almost has no influence on viscosity when amplitude exceeds 32 μm. Within high shear rate region, the viscosity values of processed material are almost equal to those of the non-processed.

#### 3.2.2. Effects of Plasticizing Pressure

**Figure 8:**
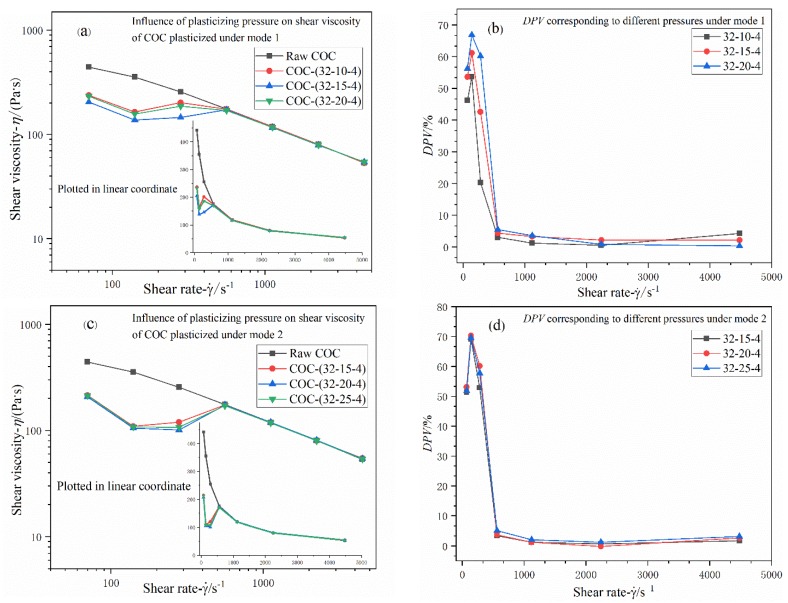
(**a**,**c**) Influence of adjusting plasticizing pressure on shear viscosity of COC plasticized in Mode 1 and Mode 2; (**b**,**d**) *DPVs* corresponding to different plasticizing pressures under two different modes. Nomenclature: ultrasonic amplitude (μm); plasticizing pressure (MPa); ultrasonic action time (s).

According to Figure 8a, the shear viscosity of COC processed under various plasticizing pressures shares the same feature when compared with that of material processed under different ultrasound amplitudes under Mode 1. Nevertheless, one remarkable difference is that the overall viscosity values of processed COC decrease with increasing plasticizing pressure in low shear region, and *DPV* reaches its maximum value (67% at 140 s^−1^) under plasticizing pressure of 20 MPa. When shear rate exceeds 560 s^−1^, the viscosity curves of raw material and modified material converged gradually; accordingly, the *DPV* remained almost in the same magnitude and invariant in the high shear rate region, as Figure 8b has demonstrated. 

Under Mode 2, the effects of plasticizing pressure on the shear viscosity of COC partake of the same characteristics as amplitude has, according to Figure 8c. Something different is that the increase of pressure affects viscosity of the raw more appreciably than amplitude does. Particularly, as shown in Figure 8d, slight separation among these curves can be discerned. The maximum *DPVs* of three parameters are all around 70% (at 140 s^−1^).

#### 3.2.3. Effects of Ultrasonic Action Time

**Figure 9:**
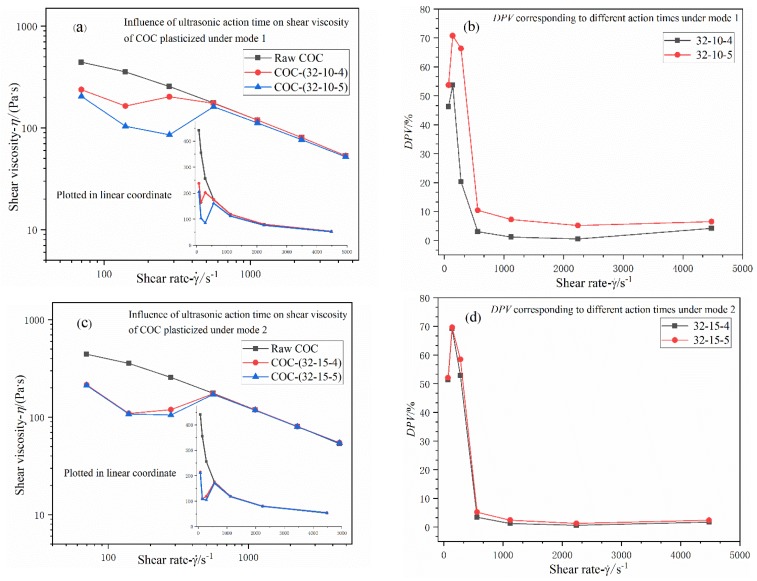
(**a**,**c**) Influence of adjusting ultrasonic action time on shear viscosity of COC plasticized under Mode 1 and Mode 2; (**b**,**d**) *DPVs* corresponding to different ultrasonic action times under two different modes. Nomenclature: ultrasonic amplitude (μm); plasticizing pressure (MPa); ultrasonic action time (s).

Under Mode 1, according to the illustration of Figure 9a,c, it seems that ultrasonic action time is the most potent factor among these ultrasonic parameters; the viscosity value at 140 s^−1^ is reduced by 71% when action time is set as 5 s; the effect of 4 s is astonishing too (decreased by 54%). Thus, it is necessary to control ultrasonic action time when ultrasound energy is applied to molding micro-sized parts. In addition, as shown in Figure 9a,c, the tendency in which viscosity changes with shear rate is quite similar to that presented in Figure 7a,c.

Time-induced effects on shear viscosity of COC in Mode 2 are as similar to regularity as what Figure 7c,d and Figure 8c,d have shown. Hence, herein, interminable depictions are not necessary. Referring to the descriptions on amplitude-induced and pressure-induced effects on viscosity of COC, time induced effects can be interpreted in the same way.

### 3.3. Effects of Ultrasonic Energy on Molecular Properties of COC

**Figure 10:**
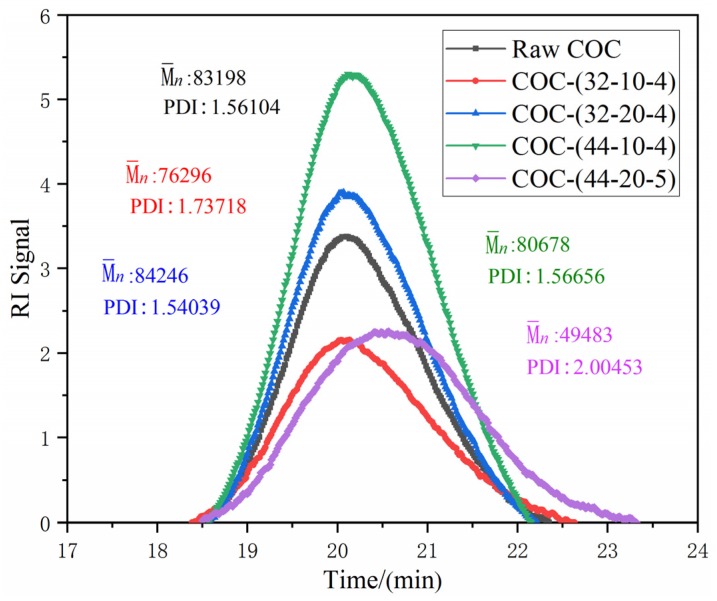
Molecular weight distribution curves of raw COC and processed COC samples under the following parameter combinations: (1) 32-10-4; (2) 32-20-4; (3) 44-10-4; (4) 44-20-5. Nomenclature: amplitude (μm); plasticizing pressure (MPa); time (s).

Based on experimental results in Section 3.2 and Section 3.3, some representative samples (prepared under Mode 1) are chosen for GPC measurement. Molecular weight and molecular distribution curves corresponding to processed COC and the raw are plotted in Figure 10. It is clear that ultrasonic vibration energy has an impact on the molecular properties of COC. According to Figure 10, except parameter combination 32-20-4 (amplitude-32 μm, pressure-20 MPa, action time-4 s), ${\bar{M}}_{n}$ values corresponding to three other process conditions are all lower than that of raw material but polymer dispersity index (*PDI*) was greater. This means that molecular chain scissions occur under these process conditions. For process condition 32-20-4, it is different from other conditions. ${\bar{M}}_{n}$ is greater than raw COC, but *PDI* is lower. Thus, polymerization between molecular chains of COC but not chain scissions is caused by ultrasound vibration energy. However, viscosity of COC processed by this condition is lower than raw COC.

### 3.4. Results of MLRA Realized by SPSS

Following the accomplishment of rheology experiment (shear viscosity measurement), relevant data needed for MLRA are selected. Table 6 and Table 7 are the variable tables designed for MLRA. The eventual results are also given in the form of a data list. To omit the randomness of analyses, the effect of each ultrasonic parameter on *DPV* corresponding to three representative shear rates (140 s^−1^, 560 s^−1^, 2240 s^−1^) was studied.

#### 3.4.1. Analytical Results for Mode 1

As to statistical results of Mode 1, it is clear that the standardized coefficient *β* of ultrasonic action time is of the biggest absolute value according to Table 8, which means that ultrasonic action time is the most powerful, which is also in line with the experimental data. Amplitude ranks the second place, but to some extent, amplitude could be considered to be as potent as time. Compared with amplitude and time, while plasticizing pressure is likely to be a less influential parameter, plasticization quality can also be affected by plasticizing pressure greatly.

#### 3.4.2. Analytical Results for Mode 2

For Mode 2, according to Table 9, the absolute value of *β* corresponding to amplitude is the biggest one, and those of plasticizing pressure and time are considerably close to each other. The aforementioned signifies that amplitude has the hugest impact on the shear viscosity of COC. Plasticizing pressure and action time are not as powerful as amplitude, but it appears that pressure and time move toward the same effect.

## 4. Discussion

In our experiments, two different modes of plasticization have been applied. In general, no matter which type of plasticization mode is applied, the shear viscosity of polymeric melts can certainly be influenced by ultrasound energy. According to the results of GPC, it can be concluded that the change of molecular properties is caused by ultrasonic energy. The decrease of ${\bar{M}}_{n}$ and the increase of *PDI* indicate that molecular chain scissions of COC have been caused by ultrasonic energy. Hence, it is certain that viscosity reduction of COC is relative to the change of molecular properties.

Moreover, there was an obvious difference between processed samples and raw COC in the low shear rate region, but almost no disparity in high shear rate can also be seen. This phenomenon could presumably be explained as follows. When the shear rate lies at a low level, shear stress exerted on the polymer melt is of a low magnitude, accordingly. Therefore, molecular chains of raw COC could not disentangle and the configuration of polymeric molecular chain is not changed. However, differently from raw COC, molecular chains of samples processed by ultrasound energy have already disentangled due to some possible physical and chemical degradation. Thus, when the shear rates are low, molecular segments of processed samples move more easily than those in raw COC. Accordingly, the viscosity values of processed samples are lower than for raw COC in the low shear rate region. With increasing shear rate, molecular chains of raw COC disentangle gradually so that the gap between the viscosity value of processed samples and that of raw COC is reduced gradually. 

As for another experimental result that the shear viscosity of processed samples shows undulant feature in low shear rate, the process volatility of ultrasonic plasticizing technology could reasonably be proposed to explain this result and the anomaly occurring during plasticizing COC with different ultrasound amplitudes. Specifically, due to volatility of the process, heterogeneous plasticization quality and uneven properties are caused. Therefore, UPMIM can be influenced by technical volatility negatively so that desirable products are difficult to be fabricated.

For the reason that two different modes have been employed, a difference in shear viscosity of processed COC is inevitable. One difference is that adjusting each ultrasonic parameter would make the shear viscosity of COC go through obvious variance under Mode 1. However, obvious discrepancy cannot be caused by parametric changes under Mode 2. Under Mode 1, polymer is exposed to ultrasonic energy during the whole plasticization process. It implies that continuous acoustic energy accumulation exists in material system; the higher input of ultrasonic power is, the more energy will be transferred into materials; as a result, some ultrasonic effects would act more intensely. Accordingly, under Mode 1, a discernible difference is caused by increasing ultrasonic parameters. In contrast to Mode 1, under Mode 2, there is no continuous ultrasonic energy accumulation in the material system on account of the swift extrusion of melted COC. Consequently, increasing each parameter is not capable of reaching the same effect as that of such an operation under Mode 1. Another difference is that the most influential ultrasonic parameters of the two modes are different. For Mode 1, ultrasound action time is the most potent at reducing the viscosity of COC. Amplitude and plasticizing pressure nearly stay at the same level. Under Mode 2, amplitude comes first, and pressure is as competent as time. Unfortunately, the reason why such a difference exists remains unknown, and further research should be done to figure out the mysteries behind this experimental phenomenon.

## 5. Conclusions

To plasticize COC successfully, an appropriate combination of ultrasound parameters is important and necessary. The interaction of ultrasound parameters results in varying plasticizing effectiveness; for instance, when ultrasonic action time is increased, amplitude and plasticizing time should be appropriately low for the purpose of obtaining non-degraded or mildly degradation COC melts. Additionally, process conditions suitable for melting COC are those which are painted blue and yellow in Table 5.

The plasticization mode has also significant influence on shear viscosity. Under Mode 1, *DPV* ranges approximately from 2% to 75%; an obvious decrease in shear viscosity is caused by increasing the magnitude of each parameter. Under Mode 2, *DPV* stays at the same level as that under mode1; However, the shear viscosity does not seem to decrease with increasing ultrasonic power input. The most influential parameter might also be determined by plasticization mode; i.e., ultrasonic action time for Mode 1 and ultrasonic amplitude for Mode 2.

For the mechanism of ultrasound induced reduction in the shear viscosity of COC, it has been proven that molecular chain scissions caused by ultrasonic energy can be accountable.

## Figures and Tables

**Table 1:** Properties of TOPAS^®^ 5013-10L COC.

Terms	Value	Test Method
Melt Mass Flow Rate/(g/10min)	48.96	ISO 1133
Solid density/(g/cm^3^)	1.02	ISO 1183
Number-average Molecular Weight/(—)	83198	GPC
Polymer dispersity index/(—)	1.56104	
Water absorption rate/(%)	0.01	ISO 62
Moisture Permeability Coefficient/(g.mm/m^2^.d)	0.03	DIN 53 122
Elastic Modulus/(MPa)	3200	ISO 527-2/1A
Tensile strength/(MPa)	46	
Elongation at Break/(%)	1.70	
Glass Transition Temperature/(°C)	134	DSC
Heat Deflection Temperature/(°C)	127 (0.45 MPa)	ISO75 part1 and part2
Transmittance/(%)	91 (*t* = 2 mm)	ISO 13468-2
Index of Refraction/(—)	1.53	ISO 13468-2

**Table 2:** Main characteristics of prototype machine.

Terms	Value
Max Clamping force/(KN)	10
Max Ejection force/(KN)	0.5
Max mold opening stroke/(mm)	150
Max ejection stroke/(mm)	10
Ultrasonic frequency/(kHz)	20
Ultrasonic amplitude/(μm)	28–52

**Table 3:** Ultrasonic amplitudes corresponding to different power inputs.

Power input/(%)	10	15	20	25	30	35	40	50	60	70
Amplitude/(μm)	28	30	32	32	34	36	36	38	38	40
Power input/(%)	75	80	85	90	95	96	97	98	99	100
Amplitude/(μm)	40	42	42	44	44	46	48	50	50	52

**Table 4:** Parameter combinations for sample preparation under Mode 1 and Mode 2.

Mode of Plasticization	Amplitude/(μm)	Pressure/(MPa)	Time/(s)
Mode 1	32	10	4
	38	10	4
	44	10	4
	32	15	4
	32	20	4
	32	10	5
Mode 2	32	15	4
	38	15	4
	44	15	4
	32	20	4
	32	25	4
	32	15	5

NB: As the raw material processed ultrasonically for 6s degraded severely in both modes, it was not suitable for preparing samples for rheology measurement.

**Table 5:** Design of the parameter combinations of the preliminary experiment and the results under Mode 1 and Mode 2.

Mode 1						Mode 2					
Time/(s)	Pressure/(MPa)	Amplitude/(μm)				Time/(s)	Pressure/(MPa)	Amplitude/(μm)			
		32	38	44	50			32	38	44	50
2	10	-	-	-	-	2	10	-	-	-	-
	15	-	-	-	-		15	-	-	-	-
	20	-	-	-	-		20	-	-	-	-
	25	-	-	-	-		25	-	-	-	-
	30	-	-	-	-		30	-	-	-	-
3	10	-	-	*	*	3	10	-	-	-	-
	15	*	*	*	*		15	-	-	-	-
	20	*	*	*	*		20	-	-	-	*
	25	*	*	*	*		25	*	*	*	*
	30	*	*	*	*		30	*	*	*	*
4	10	*	*	*	*	4	10	-	-	-	-
	15	*	*	*	*		15	*	*	*	*
	20	*	*	*	*		20	*	*	*	*
	25	*	*	*	*		25	*	*	*	*
	30	*	*	*	*		30	*	*	*	*
5	10	*	*	*	*	5	10	-	-	-	-
	15	*	*	*	*		15	*	*	*	*
	20	*	*	*	*		20	*	*	*	*
	25	*	*	*	*		25	*	*	*	*
	30	*	*	*	*		30	*	*	*	*
6	10	*	*	*	*	6	10	*	*	*	*
	15	*	*	*	*		15	*	*	*	*
	20	*	*	*	*		20	*	*	*	*
	25	*	*	*	*		25	*	*	*	*
	30	*	*	*	*		30	*	*	*	*
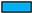 No degradation		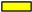 Mild degradation				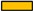 Moderate degradation		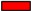 Severe degradation			

NB: The symbol “**-**“ indicates parameter combinations in which COC can not be plasticized; symbol “*” indicates those in which COC can be plasticized.

**Table 6:** Variables involved in MLRA for mode 1 and their values.

Temperature/(°C)			240		
$\mathrm{Shear}\;\mathrm{Rate}-\dot{\gamma }$/(s^−1^)			140	560	2240
Amplitude/(μm)	Plasticizing Pressure/(MPa)	Time/(s)	*DPV1*/(%)	*DPV2*/(%)	*DPV3*/(%)
32	10	4	53.67	3.01	0.51
32	15	4	61.14	4.32	2.18
32	20	4	66.82	5.48	0.82
38	10	4	62.10	6.12	2.25
44	10	4	48.95	9.25	4.20
32	10	5	70.77	10.42	5.14

**Table 7:** Variables involved in MLRA for mode 2 and their values.

Temperature/(°C)			240		
$\mathrm{Shear}\;\mathrm{Rate}-\dot{\gamma }$/(s^−1^)			140	560	2240
Amplitude/(μm)	Plasticizing Pressure/(MPa)	Time/(s)	*DPV1*/(%)	*DPV2*/(%)	*DPV3*/(%)
32	15	4	69.16	3.39	0.66
32	20	4	70.28	3.74	−0.19
32	25	4	69.57	5.09	1.21
38	15	4	73.63	5.43	2.00
44	15	4	73.00	5.65	1.93
32	15	5	69.64	5.20	1.29

**Table 8:** MLRA results for Mode 1.

Shear Rate/(s^−1^)	Ultrasonic Parameter	*β*
140	Amplitude	0.491
	Plasticizing pressure	−0.228
	Time	**0.693**
560	Amplitude	0.907
	Plasticizing pressure	0.362
	Time	**1.054**
2240	Amplitude	0.722
	Plasticizing pressure	0.099
	Time	**0.942**

**Table 9:** MLRA results for Mode 2.

Shear Rate/(s^−1^)	Ultrasonic Parameter	*β*
140	Amplitude	**0.796**
	Plasticizing pressure	−0.089
	Time	−0.108
560	Amplitude	**1.100**
	Plasticizing pressure	0.602
	Time	0.732
2240	Amplitude	**0.861**
	Plasticizing pressure	0.126
	Time	0.372
